# Inhibitory Effects and Mechanisms of Flavonoids in Sea Buckthornon (*Hippophae rhamnoides* L.) on *Helicobacter pylori*

**DOI:** 10.3390/foods14233995

**Published:** 2025-11-21

**Authors:** Huicui Liu, Kunhao Xie, Yueyue Rui, Shulin Wang

**Affiliations:** College of Agriculture and Animal Husbandry, Qinghai University, Xining 810016, China; huicuill@163.com (H.L.); xiekunhao@163.com (K.X.); 18405580802@163.com (Y.R.)

**Keywords:** flavonoids in Sea Buckthornon, *Helicobacter pylori*, inhibitory effects

## Abstract

This study employed wild sea buckthorn (*Hippophae rhamnoides* L.) fruits harvested in Qinghai Province as experimental material. Following compositional analysis of their flavonoids, the antibacterial efficacy and mechanistic pathways of flavonoids in sea buckthorn against *Helicobacter pylori (H. pylori)* were systematically examined through in vitro and animal model experiments. The results showed that the main flavonoids in sea buckthorn were rutin, quercetin-3-O-glucoside, quercetin, catechin, isorhamnetin, kaempferol-3-O-glucoside, kaempferol and epicatechin. Among them, quercetin, isorhamnetin and kaempferol had strong inhibitory activity against *H. pylori.* In vitro, sea buckthorn flavonoids inhibited the growth of *H. pylori* through multiple mechanisms: inducing morphological transformation from rod-shaped to spherical bacteria, promoting cell shrinkage and rupture, disrupting the cell membrane to cause leakage of intracellular macromolecules, increasing membrane permeability, and suppressing urease activity. Sea buckthorn flavonoids exert therapeutic effects on *H. pylori*-infected mice through multiple mechanisms, including the alleviation of gastric mucosal inflammation via the Nuclear Factor KappaB (NF-κB) signaling pathway, the down-regulation of gastrin-17 (GAS17) to suppress gastric acid production, and the up-regulation of epidermal growth factor (EGF) expression to promote gastric mucosal repair and modulate the composition of gastric microbiota. This study systematically elucidated the anti-*H. pylori* activity and antibacterial mechanisms of flavonoids derived from sea buckthorn fruits, providing a theoretical basis for the screening of natural antibacterial agents from this plant source.

## 1. Introduction

*H. pylori*, a Gram-negative bacterium, colonizes the human gastric mucosa. It was first isolated and identified by Warren and Marshall in 1982 and was subsequently confirmed as the causative agent of peptic ulcers in humans [1]. Further research has demonstrated that infection with this pathogen not only leads to gastric and duodenal ulcers but also contributes to the development of gastric lymphoma and gastric cancer over time. Although *H. pylori* is not the sole etiological factor for gastritis or peptic ulcers, it is responsible for over 90% of duodenal ulcers and approximately 80% of gastric ulcers. Furthermore, more than 60% of patients with chronic gastritis are infected with *H. pylori* [2,3,4]. It has been reported that *H. pylori* colonizes the gastric epithelial cells of at least half of the global population [5]. Statistical data indicate that the infection rate in China reaches as high as 56.2% [6]. *H. pylori* primarily induces inflammatory responses in host cells through virulence factors such as cytotoxin-associated gene A (CagA) and vacuolating cytotoxin A (VacA) [5]. The CagA protein enhances inflammatory responses by activating the NF-κB signaling pathway in the host, thereby promoting the development of precancerous lesions associated with gastric cancer [7], whereas VacA leads to the loss of mitochondrial membrane potential and initiates the process of apoptosis [8]. Currently, the treatment of *H. pylori* primarily relies on the administration of antibiotics in combination with proton pump inhibitors (PPIs), such as triple therapy consisting of clarithromycin, amoxicillin, and a PPI, or quadruple therapy involving bismuth, metronidazole, tetracycline, and a PPI [9]. Quadruple therapy with bismuth-based regimens, as well as concomitant therapy combining tinidazole with clarithromycin, has been shown to achieve eradication rates ranging from 85% to 99% [10]. However, antibiotic treatment is associated with a reduction in the diversity of the intestinal microbiota, and prolonged antibiotic use may lead to dysbiosis of the gut microbiota, potentially exacerbating the host’s inflammatory response [11]. Therefore, the development of novel antibacterial agents, such as natural compounds, has become increasingly urgent [12,13].

At present, the antibacterial activity of natural compounds against *H. pylori* has been widely reported. Sosa et al. reported that olive oil and hydroxytyrosol exert inhibitory effects on *H. pylori* by compromising the integrity of the bacterial cell membrane [14]. Trung et al. demonstrated that apigenin, a component of hibiscus extract, inhibits *H. pylori* through suppression of biofilm formation, while myricetin exhibits a potent inhibitory effect on urease activity [15]. The combination of natural compounds with antibiotics has been shown to enhance the efficacy of antibiotic treatment. For example, Bhattamisra’s research showed that geraniol combined with antibiotics has an additive effect on *H. pylori*, and when geraniol was used in combination with antibiotics against *H. pylori*, the doses of amoxicillin and clarithromycin can be reduced by 32 times [16]. Collectively, these findings suggest that natural products may inhibit *H. pylori* through mechanisms such as disruption of bacterial membrane integrity and suppression of key enzymatic activities.

Our team’s preliminary research has demonstrated that malic acid, oxalic acid, and tartaric acid present in sea buckthorn exhibit significant anti-*H. pylori* activity. These three organic acids are capable of inhibiting a range of essential *H. pylori* genes, including urease genes (ureA, ureB), virulence genes (VacA, CagA), replication genes (dnaE, dnaN, dnaQ), transcription genes (rpoA, rpoD, rpoN), motility genes (flhA, flaA, flgE), adhesion genes (alpA, alpB, hpaA, hpaZ), and outer membrane protein genes (BabA). Furthermore, they possess the ability to suppress the expression of VacA and CagA proteins, thereby mitigating the inflammatory response induced by *H. pylori* infection [17]. Meanwhile, preliminary exploratory studies conducted by our team have indicated that various extracts of sea buckthorn-including ethyl acetate extract, water extract, ethanol extract—as well as organic acids and catechin compounds exhibit antibacterial properties. Although the aforementioned studies indicate that sea buckthorn extract exhibits inhibitory activity against *H.pylori*, it is currently still used only as an adjunctive therapy or functional food and has not yet been approved for clinical use as a pharmaceutical agent. Moreover, the potential antibacterial effects of flavonoids in sea buckthorn on *H. pylori* in vitro, the underlying mechanisms of such effects, and their therapeutic efficacy in vivo against *H. pylori* infection remain unexplored. Therefore, in this study, the antibacterial activity and potential mechanisms of flavonoids in sea buckthorn against *H. pylori* were systematically evaluated using in vitro antibacterial assays combined with an *H. pylori*-infected mouse model. This research would provide a theoretical basis and novel strategies for the efficient development and utilization of sea buckthorn resources.

## 2. Methods

### 2.1. Chemicals and Reagents

Quercetin, isorhamnetin, kaempferol, rutin, polymyxin B, amphotericin B, vancomycin hydrochloride, trimethoprim were purchased from Beijing Soleibao Biotechnology Co., Ltd. (Beijing, China). The reagents used for *H. pylori* culture including solid medium, brucella broth, *H. pylori* additive were obtained from Haibo Biotechnology Co., Ltd. (Qingdao, China). Defibrated sheep blood was purchased from Henan Jushi Biology Co., Ltd. (Zhengzhou, China). Other chemicals and reagents used in this study were of analytical grade.

### 2.2. Materials and Strains

The fresh and undamaged fruits of wild sea buckthorn were collected in Haidong City, Qinghai Province, China (36.37° N, 102.27° E, altitude 3000–3300 m). The *H. pylori* ATCC 43504 strain was purchased from Shangcheng Beina Chuanglian Biotechnology Co., Ltd. (Xinyang, China). The culture method of *H. pylori* was carried out according to the method of Gao et al. [17]. *H. pylori* was cultured on Columbia blood agar supplemented with 10% fetal bovine serum and 1% mixed antibiotics (polymyxin, vancomycin, amphotericin B, trimethoprim). The solid culture was maintained at 37 °C in a microaerophilic environment (85% N_2_, 10% CO_2_, 5% O_2_) for 72 h.

### 2.3. Extraction and Purification of Sea Buckthorn Flavonoids

The sea buckthorn fruits were carefully selected and freed from impurities such as leaves and branches. Subsequently, the fruits were dried in a hot air oven at 50 °C for 24 h, subsequently ground using a high-speed pulverizer operating at 28,000 rpm, and then sieved through a 60-mesh sieve for further use [18]. An appropriate quantity of the resulting powder was weighed, and petroleum ether was added at a solid-to-liquid ratio of 1:10 g/mL. The mixture was allowed to stand for 24 h for defatting purposes, after which it was filtered and transferred to an oven maintained at 50 °C until complete evaporation of the petroleum ether occurred. This process yielded the defatted sea buckthorn powder [18]. The crude extract of sea buckthorn flavonoids was prepared by adapting the method of Xie and subsequently stored at 4 °C in a refrigerator. Following vacuum concentration and freeze-drying, the final crude extract of sea buckthorn flavonoids was obtained [19]. The purification of sea buckthorn flavonoids was conducted following the methodology of Zhao [20], utilizing AB-8 macroporous adsorption resin. The process parameters included a sample loading concentration of 2 mg/mL, a loading flow rate of 1.5 mL/min, a total loading volume of 70 mL, an elution flow rate of 2 mL/min, and an eluent volume of 100 mL [20].

### 2.4. Flavonoids of Sea Buckthorn Composition Analysis

The flavonoid composition in sea buckthorn was analyzed using an UHPLC–Orbitrap-MS system. Qualitative and quantitative detection of flavonoid extracts in sea buckthorn were achieved by comparing their retention times, mass-to-charge ratios, and other relevant data with those of corresponding standard substances [21,22,23].

### 2.5. Identification of H. pylori

Following a 72 h culture of *H. pylori* on solid medium, bacterial growth and colony morphology were examined through visual inspection. Colonies were gently harvested from the culture medium and transferred onto urease test strips. The enzymatic reaction was evaluated after a 5 min incubation period, wherein retention of the original color denoted a urease-negative phenotype, while development of a crimson hue confirmed urease positivity. In parallel, catalase activity was determined by introducing 2% (*v*/*v*) hydrogen peroxide solution to 72 h cultures; immediate effervescence was interpreted as indicative of *H. pylori* viability. In addition, *H. pylori* was stained according to the instructions of the Gram staining kit and observed under a microscope. If the bacteria appeared red, it indicates that the bacteria were Gram-negative. Finally, the isolated bacterial strains were identified through molecular biological analysis employing 16S rDNA sequencing technology.

### 2.6. Assessment of the Antibacterial Activity of H. pylori In Vitro

#### 2.6.1. Inhibition Zone Experiment

The antibacterial activity of flavonoids was evaluated using the paper disk method. Specifically, 100 μL of the bacterial suspension was uniformly spread across the surface of the *H. pylori* solid medium. The medium was then left at room temperature for 20 min to allow the surface to become slightly dry. Subsequently, a sterile blank antibiotic sensitivity paper disk with a diameter of 6 mm was carefully immersed in the sample solution at a concentration of 20 mg/mL using sterile tweezers (three disks were tested in each group). These impregnated filter paper disks were placed at equidistant intervals on the agar plate. The plate was then inverted and incubated at 37 °C for 48 h. Finally, the diameter of the inhibition zone was measured using the cross method with a vernier caliper.

#### 2.6.2. Determination of the Minimum Inhibitory Concentration (MIC)

The MIC of flavonoids was determined using the broth microdilution method [24]. The flavonoid standard was dissolved in DMSO to prepare a stock solution with a concentration of 20 mg/mL. A mixed standard solution was obtained by combining quercetin, isorhamnetin, kaempferol and rutin at equal volumes. Serial dilutions of the 20 mg/mL flavonoid stock solution were performed in a 96-well microtiter plate using Brucella broth, resulting in final concentrations of 10, 5, 2.5, 1.25, 0.625, 0.313, 0.156, and 0.078 mg/mL. Subsequently, 50 μL of bacterial suspension and 50 μL of culture broth were added to each well, yielding a total reaction volume of 200 μL per well. The bacterial suspension without the addition of flavonoid standard served as the blank control. Following thorough mixing, the 96-well plate was placed in a sealed incubator and incubated at 37 °C for 48 h. The MIC was defined as the lowest concentration corresponding to the first well exhibiting no visible turbidity.

#### 2.6.3. Growth Inhibition Assay

The culture medium supplemented with flavonoids at concentrations of 0, 1/4 MIC, 1/2 MIC, MIC, and 2 MIC was inoculated with *H. pylori* in a 96-well plate and incubated at 37 °C for 48 h. Following incubation, the optical density at 600 nm was measured [17,25].

#### 2.6.4. Determination of Cell Membrane Permeability

The effect of flavonoids extracted from sea buckthorn on the outer membrane permeability of *H. pylori* cells was evaluated using the fluorescent probe N-phenyl-1-naphthylamine (NPN) [26]. Briefly, *H. pylori* was cultured in a 96-well microplate in the presence of varying concentrations of flavonoids (0, 1/4 MIC, 1/2 MIC, MIC, and 2 MIC). Following the addition of 20 μL of NPN solution, the samples were incubated at 37 °C for 1 h. Fluorescence intensity was subsequently measured using a microplate reader with excitation and emission wavelengths set at 350 nm and 420 nm, respectively.

#### 2.6.5. Determination of Cell Content Leakage

Flavonoids were added to the medium containing *H. pylori* to achieve the final concentrations 0, 1/4 MIC, 1/2 MIC, MIC, 2 MIC and 4 MIC, respectively, and it was incubated in a constant temperature incubator at 37 °C for 12 h. Then, the bacterial suspensions from the various treatment groups were subjected to centrifugation, and the resulting supernatants were collected and analyzed at 260 nm and 280 nm wavelength, respectively.

#### 2.6.6. Urease Activity Assay

*H. pylori* was cultured for 48 h, and the bacterial suspension was subsequently prepared using phosphate-buffered saline. The bacterial suspension was then mixed with varying concentrations of flavonoids (0, 1/4 MIC, 1/2 MIC, MIC, 2 MIC, and 4 MIC), followed by incubation at 37 °C for 2 h. Afterward, a mixture containing 0.02% cresol red, 0.1% EDTA, and 1.5% urea was added, and the solution was further incubated at 37 °C for 20 min. The absorbance was then measured at 590 nm [27].

#### 2.6.7. Morphological Observation by Scanning Electron Microscope (SEM)

*H. pylori* morphological analysis was executed after exposure to the inhibitory of quercetin, isorhamnetin, kaempferol, mixed standards and sea buckthorn flavonoids at the concentration of MIC using a SEM (Resolution: 0.6 nm (15 kV) and 0.7 nm (1 kV); JSM-7900F, Japan Electronics Co., Ltd. (Tokyo, Japan)) [17,24].

### 2.7. Assessment of the Antibacterial Activity of H. pylori In Vivo

#### 2.7.1. Animals and Treatments

**Figure 1:**
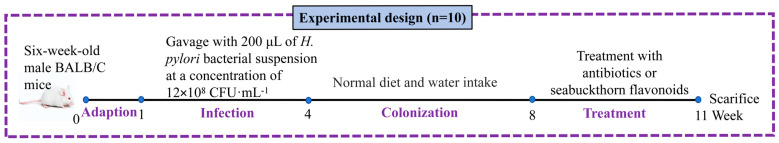
The experimental design for evaluating the effects of sea buckthorn flavonoids on *H. pylori*-infected mice.

Female specific pathogen-free BALB/C mice (6 weeks old) were provided and feeding by Beijing Weishang Lide Biotechnology Co., Ltd. (Beijing, China). The experimental design for evaluating the effects of sea buckthorn flavonoids on *H. pylori*-infected mice was depicted in Figure 1.

After 1 week of adaptive culture, the mice were randomly assigned to eight groups (*n* = 10): NC, MC, PC, LF, MF, HF, MS and Q groups. The details of these groups were provided below: NC, the mice were orally administered with 200 μL of sterile HPM-S broth once every 2 days for three consecutive weeks, followed by oral gavage with 200 μL of sterile water every day for seven consecutive weeks. Groups MC, PC, LF, MF, HF, MS and Q, the mice were orally administered with 200 μL of *H. pylori* (12 × 10^8^ CFU·mL^−1^) at two-day intervals for three consecutive weeks to achieve infection. Then, the mice in groups MC, PC, LF, MF, HF, MS and Q were given normal diet and water for three consecutive weeks to *H. pylori* colonization. After *H. pylori* colonization, the mice in groups MC, PC, LF, MF, HF, MS and Q were orally administered with 200 μL of corresponding testing solutions (MC: sterile water; PC: a triple therapy (15.17 mg/mL Metronidazole, 22.75 mg/mL amoxicillin, 0.3034 mg/mL omeprazole); LF: 100 mg/kg sea buckthorn flavonoids; MF: 200 mg/kg sea buckthorn flavonoids; HF: 300 mg/kg sea buckthorn flavonoids; MS: mixed standard solution of quercetin, isorhamnetin and kaempferol at a ratio of 10:5:1 at a dose of 50 mg/kg/d; Q: 50 mg/kg/d quercetin) for three consecutive weeks. The mice were sacrificed by cervical dislocation after treatment. Gastric mucosal samples and gastric contents were collected and frozen in liquid nitrogen and kept at −80 °C until analysis.

#### 2.7.2. Hematoxylin and Eosin Staining (H&E) of Gastric Tissue

The H&E was carried out according to previous studies [28].

#### 2.7.3. Determination of Inflammatory and Growth Factors in Mice Serum

The concentrations of EGF, TNF-α, CXCL1, IL-1β, IL-2, G17, and GAS17 in mouse serum were determined using ELISA kits, with specific procedures performed in accordance with the manufacturer’s instructions.

#### 2.7.4. RT-qPCR Assay

Total RNA was extracted from 50 mg of gastric tissue samples, followed by reverse transcription using a commercial reverse transcription kit to synthesize cDNA. A 10 μL qPCR reaction mixture was prepared containing 5 μL of SYBR qPCR SuperMix Plus (Sichuan Lanyun Biology Co., Ltd. (Chengdu, China)), 4 μL of the synthesized cDNA, and 0.5 μL of each specific upstream and downstream primer. Real-time quantitative PCR was performed on a SLAN-96S RT-qPCR system. The relative mRNA expression level of the target gene was normalized to the internal reference gene β-actin and calculated using the 2^−ΔΔCt^ method [29]. The primer sequences are listed in Table S1.

#### 2.7.5. Microbiota Analysis by 16S rDNA in Gastric Content

The 16S rDNA sequencing and analysis of the gastric contents in mice were performed by Beijing Qikexin Biotechnology Co., Ltd. (Beijing, China).

### 2.8. Statistical Analysis

The data were analyzed and presented as means ± standard deviation. The IBM SPSS 22.0 software was used to perform the statistical analysis. A sample difference with *p* ≤ 0.05 was considered significant. Data were analyzed using the Prism 10.1.2 (GraphPad, San Diego, CA, USA) software. Graph bars with different letters on the top indicate significant differences (*p* < 0.05) using the Tukey–Kramer method.

## 3. Results

### 3.1. Flavonoids of Sea Buckthorn Composition

As presented in Table 1, rutin, quercetin-3-O-glucoside, 3,4-Dihydroxybenzoic acid, quercetin, catechin, isorhamnetin, kaempferol-3-O-glucoside, kaempferol, and epicatechin are the primary constituents of sea buckthorn flavonoids. Among these compounds, rutin, quercetin-3-O-glucoside, 3,4-dihydroxybenzoic acid, and quercetin are present at relatively higher concentrations.

### 3.2. Culture and Identification of H. pylori

**Figure 2:**
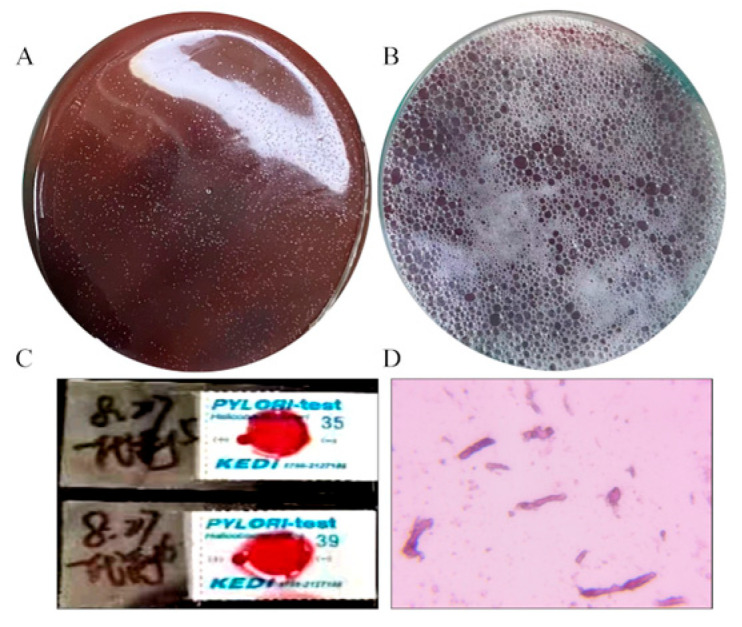
Cultivation and identification of *H. pylori*. (**A**) The colony morphology of *H. pylori*, (**B**) the catalase activity of *H. pylori*, (**C**) the result of the urease test strip for detecting *H. pylori*, (**D**) *H. pylori* Gram staining.

The results of the *H. pylori* culture and identification were shown in Figure 2. After 36 h of solid culture, the colony morphology of *H. pylori* was small, semi-transparent and needle-point-like, with a diameter of about 1 mm (Figure 2A). The surface of the culture medium was frosted, which was in line with the characteristics of *H. pylori* colonies. *H. pylori* possesses highly active catalase, which helps it eliminate reactive oxygen species produced by host immune cells and avoid oxidative damage to itself. This characteristic is one of the important identification criteria for it. As shown in Figure 2B, when 3% hydrogen peroxide was dropped onto the solid culture medium, a large number of bubbles rapidly form on the surface of the colonies, suggesting the presence of catalase, which is in line with the characteristics of *H. pylori* colonies. Meanwhile, the cultured bacteria were tested positive by the *H. pylori* urease test strip, indicating that the cultured bacteria have urease activity, which is also in line with the characteristics of *H. pylori* (Figure 2C). The Gram staining result indicated that *H. pylori* is a Gram-negative bacterium (Figure 2D). Moreover, the 16S rDNA sequences of the *H. pylori* strains were extracted and compared with the ribosomal database of NCBI (Table 1). We found that the bacteria had the highest similarity to the 16S rDNA of the ATCC43504 type *H. pylori* strain (Table 2). Together with the above findings, the bacteria we cultivated were indeed of the *H. pylori*.

### 3.3. The Antibacterial Effect of Flavonoids in Sea Buckthorn on H. pylori In Vitro

#### 3.3.1. The Diameter of the Antibacterial Zone and MIC

**Figure 3:**
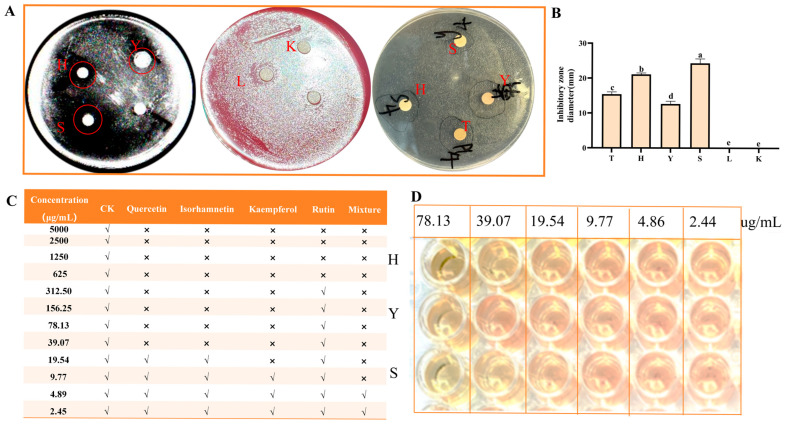
Determination of inhibition zone diameter and MIC of *H. pylori*. (**A**) Antibacterial zone; (**B**) the diameter of inhibition zone; (**C**) MIC determination; (**D**) MIC determination of quercetin, isorhamnetin, and kaempferol. K is the control; T is sea buckthorn flavonoids; H is quercetin; Y is isorhamnetin; S is kaempferol; L is rutin. “√” indicates turbidity visible to the naked eye; “×” indicates no obvious turbidity. Bars labeled with different letters (a, b, c, d, e) denote statistically significant differences at *p* ≤ 0.05, as determined by the Tukey–Kramer method.

As shown in Figure 3A, except for the control group and the rutin standard group, the other three flavonoid treatment groups had inhibition zones. Compared with the sea buckthorn flavonoid extract, the inhibition zones in the quercetin and kaempferol standard groups were larger. Compared with other flavonoids, the inhibition zones of kaempferol (24.33 ± 1.26 mm), quercetin (21.12 ± 0.38 mm), and isoquercitrin (12.67 ± 0.76 mm) were larger (Figure 3B). Among them, the inhibition effects of kaempferol and quercetin were stronger, while the inhibition zone of isoquercitrin was smaller. As shown in Figure 3C, the MIC of quercetin, isorhamnetin, kaempferol, rutin, and the mixed standard were 39.07, 39.07, 19.54, 625, and 9.77 μg/mL, respectively. The four standard substances showed a synergistic effect when mixed. The order of antibacterial activity against *H. pylori* of the four flavonoids was: kaempferol > quercetin > isorhamnetin > rutin. The MIC test results indicated that kaempferol, quercetin, and isorhamnetin had the best antibacterial effects, which were basically consistent with the results of the inhibition zone experiment. Therefore, quercetin, isorhamnetin, and kaempferol were selected for further study.

#### 3.3.2. The Inhibition Rate of Flavonoids in Sea Buckthorn on *H. pylori*

**Figure 4:**
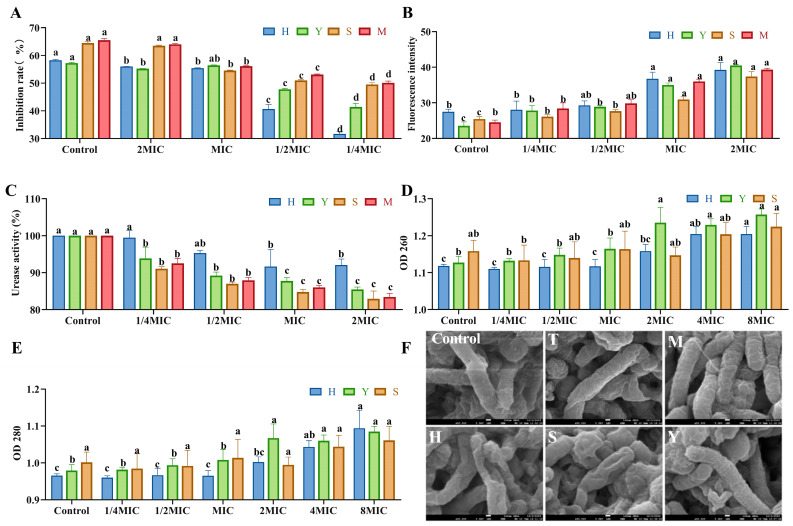
Effect of sea buckthorn flavonoids on the inhibition rate (**A**), outer membrane permeability (**B**), urease activity (**C**), membrane integrity (**D**,**E**), and morphology (**F**), magnification times: 50,000 times; scale bar: 100 nm) of *H. pylori*. In Figure 4F, T is sea buckthorn flavonoids; M is mixed standard; H is quercetin; S is kaempferol; Y is isorhamnetin. Significant difference annotations indicate comparisons between different concentrations within the same group. Bars labeled with different letters (a, b, c, d) denote statistically significant differences at *p* ≤ 0.05, as determined by the Tukey–Kramer method.

As illustrated in Figure 4A, the inhibitory rates of kaempferol, quercetin, isorhamnetin, and the mixed standard compounds against *H. pylori* increased proportionally with the increasing concentration. Compared with the 1/4 MIC treatment group, the inhibitory rates of the MIC, 2 MIC, and 4 MIC treatment groups against *H. pylori* were significantly higher (*p* < 0.05), with all rates exceeding 50%. This indicates that the inhibitory effect of sea buckthorn flavonoids on *H. pylori* was pronounced at concentrations greater than the MIC.

#### 3.3.3. The Effect of Sea Buckthorn Flavonoids on the Extracellular Membrane Permeability of *H. pylori*

As shown in Figure 4B, the extracellular membrane fluorescence intensity of quercetin, isorhamnetin, kaempferol and the mixed standard samples increased gradually in a dose-dependent manner with the increase in concentration. Compared with the control group, the extracellular membrane fluorescence intensity of *H. pylori* cells in the MIC and 2 MIC treatment groups was significantly enhanced (*p* < 0.05). Escandón et al. suggested that one possible mechanism of the antibacterial effect of flavonoids is to disrupt the cell membrane by targeting phospholipids [30]. Based on this, we speculated that the treatment of quercetin, isorhamnetin and kaempferol can increase the permeability of the extracellular membrane of *H. pylori* by destroying it, thereby achieving an antibacterial effect.

#### 3.3.4. Inhibitory Effect on Urease Activity

The strong urease activity of *H. pylori* enables it to resist the adverse effects of the acidic environment in the stomach by generating ammonia substances, yet this same activity also contributes to damage of the gastric mucosa [31]. As illustrated in Figure 4C, the urease activity in the MIC and 2 MIC treatment groups was significantly lower than that in the blank control group (*p* < 0.05). Among them, the inhibitory effects of isorhamnetin, kaempferol and the mixed standard were better. Urease activity was reduced by 85.45%, 82.94% and 83.43% at the concentrations of 2 MIC for isorhamnetin, kaempferol and the mixed standard, respectively. Quercetin, isorhamnetin, and kaempferol may inhibit urease activity by disrupting protein conformation [32,33].

#### 3.3.5. Effects on Leakage of Bacterial Cell Contents

The effects of sea buckthorn flavonoid substances such as quercetin, kaempferol, and isoquercitrin on the leakage of cell contents are shown in Figure 4D,E. The leakage of intracellular nucleic acids was characterized by the absorbance at 260 nm, and the leakage of proteins was characterized by the absorbance at 280 nm. The concentrations of proteins and nucleic acids increased progressively with the increasing concentrations of the treatment groups. Compared with the control group, nucleic acid leakage from *H. pylori* cells significantly increased when the concentration of quercetin was 4MIC and that of isorhamnetin was 2MIC. Furthermore, protein leakage from *H. pylori* cells significantly increased when quercetin was at 4MIC and isorhamnetin at 8MIC. In contrast, kaempferol did not significantly affect either nucleic acid or protein leakage in *H. pylori* cells.

#### 3.3.6. Effects of Flavonoids in Sea Buckthorn on the Structure of *H. pylori*

As illustrated in Figure 4F, the majority of bacterial cells in the control group exhibit an intact rod-shaped morphology with smooth surfaces and well-defined boundaries. In contrast, in the groups treated with isorhamnetin, kaempferol, sea-buckthorn flavonoid extract, and the mixed standard, a morphological transformation of *H. pylori* from rod-shaped to spherical forms was observed. Additionally, surface shrinkage and structural alterations in the bacterial cells were evident. These findings clearly indicate that the bioactive flavonoid in sea-buckthorn can inhibit *H. pylori* growth by inducing morphological changes and disrupting the integrity of the cell membrane and cellular architecture.

### 3.4. The Effect and Mechanism of Sea Buckthorn Flavonoids on Inhibiting H. pylori Infection and Colonization In Vivo

#### 3.4.1. Changes in the Body Weight of Mice

**Figure 5:**
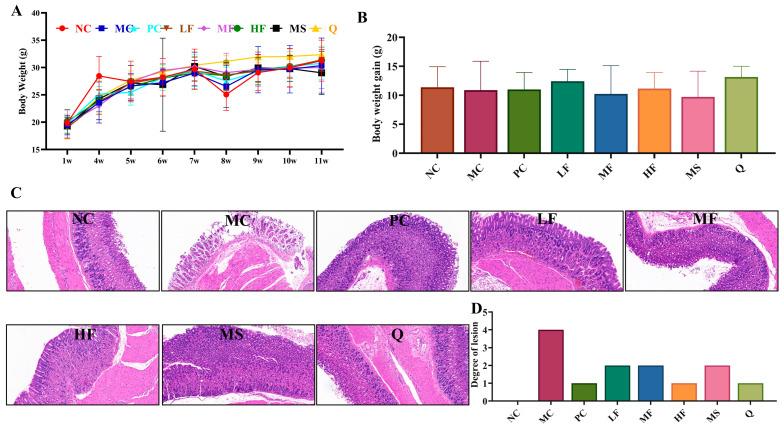
The effect of sea buckthorn flavonoids on the body weight and inflammatory response in the gastric tissues of mice infected with *H. pylori.* Body weight changes (**A**), body weight gain (**B**), gastric tissue H&E staining (magnification times: 200 times, (**C**)) and lesion degree of mice (**D**).

As shown in Figure 5A, the body weight of all groups of mice showed an overall up-ward trend. In the eighth week, except for the NC group, the body weight of the other groups decreased to varying degrees. It is speculated that the colonization of *H. pylori* led to the occurrence of gastric inflammation, thereby causing the weight loss. After the 11th week of feeding, there was no significant difference in the body weight gain of mice in each group (Figure 5B, *p* > 0.05).

#### 3.4.2. The Effect of Sea Buckthorn Flavonoids on the Inflammatory Response in the Gastric Tissues of Mice Infected with *H. pylori*

As illustrated in Figure 5 C,D, the gastric mucosal cells in the NC group exhibited a well-organized arrangement, with no evident inflammatory cell infiltration observed in the submucosal layer. In contrast, the MC group displayed degeneration and necrosis of gastric mucosal cells, characterized by a loose and edematous structure, along with a significant infiltration of inflammatory cells in the lamina propria (grade 4 inflammation). Group PC specimens showed well-maintained mucosal integrity with focal submucosal inflammatory microinfiltrates (grade 1 inflammation). LF group histological sections displayed physiologically normal mucosal stratification without evidence of cytopathic effects (grade 2 inflammation). MF analysis revealed mild epithelial detachment with moderate inflammatory cell infiltration within the lamina propria (grade 2 inflammation). Mirroring NC group findings, group HF presented with properly aligned mucosal architecture showing no detectable signs of inflammation (grade 1 inflammation). Group MS displayed mild epithelial desquamation combined with subtle lamina propria congestion and sparse inflammatory cell presence (grade 2 inflammation). Group Q demonstrated mild epithelial detachment concurrent with limited inflammatory infiltration in the lamina propria (grade 1 inflammation). It can be seen that each group of sea buckthorn flavonoids treatment has a certain therapeutic effect on gastric inflammation caused by *H. pylori* infection.

#### 3.4.3. The Effect of Sea Buckthorn Flavonoids on the Levels of Inflammatory Factors and Growth Factors in the Serum of Mice

**Figure 6:**
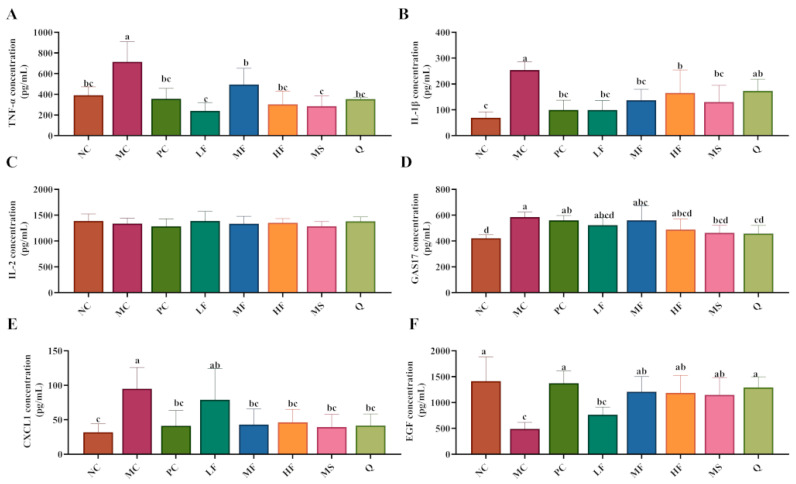
The effect of sea buckthorn flavonoids on the levels of inflammatory factors and growth factors in the serum of *H. pylori* infected mice. (**A**) Serum TNF-α level; (**B**) Serum IL-1β level; (**C**) Serum IL-2 level; (**D**) Serum GAS17 level; (**E**) Serum CXCL1 level; (**F**) Serum EGF level. Bars labeled with different letters (a, b, c, d) denote statistically significant differences at *p* ≤ 0.05, as determined by the Tukey-Kramer method.

As shown in Figure 6A,B, compared with the NC group, the serum levels of TNF-α (no significant difference, *p* > 0.05) and IL-1β (significant difference, *p* < 0.05) were elevated in the MC group. Following treatment with PC, LF, MF, HF, and MS, the serum levels of TNF-α and IL-1β were significantly reduced (*p* < 0.05), with PC, LF, MF, and HF demonstrating more pronounced therapeutic effects. However, no significant differences were observed in serum IL-2 levels across all groups (*p* > 0.05; Figure 6C). As illustrated in Figure 6D–F, compared with the NC group, the serum levels of GAS17 and CXCL1 were significantly increased in the MC group (*p* < 0.05), whereas the level of EGF was markedly decreased (*p* < 0.05). Compared with the MC group, MS and Q significantly reduced serum GAS17 levels in *H. pylori*-infected mice, while PC, MF, HF, MS, and Q significantly lowered serum CXCL1 levels and increased EGF expression (*p* < 0.05). Collectively, these findings suggest that sea buckthorn flavonoids may exert anti-inflammatory effects by reducing the serum levels of TNF-α and IL-1β in *H. pylori*-infected mice. Additionally, sea buckthorn flavonoids may inhibit excessive gastric acid secretion by suppressing the production of GAS17 and CXCL1 and promote mucosal repair through up-regulation of EGF expression.

#### 3.4.4. The Regulatory Effects of Sea Buckthorn Flavonoids on the mRNA Expression of Inflammatory and Growth Factors in the Gastric Tissues of Mice

**Figure 7:**
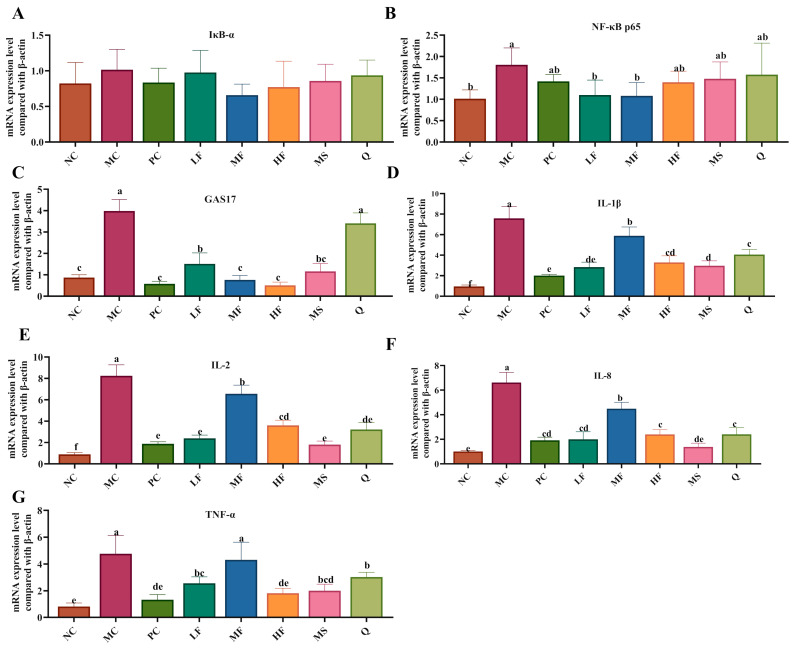
Effects of sea buckthorn flavonoids on *H. pylori*-related gene expression. (**A**) The mRNA expression of IκB-α. (**B**) The mRNA expression of NF-κB p65. (**C**) The mRNA expression of GAS17. (**D**) The mRNA expression of IL-1β. (**E**) The mRNA expression of IL-2. (**F**) The mRNA expression of IL-8. (**G**) The mRNA expression of TNF-α. Bars labeled with different letters (a, b, c, d, e) denote statistically significant differences at *p* ≤ 0.05, as determined by the Tukey-Kramer method.

As illustrated in Figure 7A, no significant differences (*p* > 0.05) were observed in the mRNA expression levels of IκB-α in the gastric tissues across the experimental groups of mice. Compared with the NC group, the mRNA expression levels of NF-κB p65, GAS17, IL-1β, IL-2, IL-8, and TNF-α in the gastric tissues of *H. pylori*-infected mice in the MC group were significantly upregulated (Figure 7B–G, *p* < 0.05). Following treatment interventions, the expression of NF-κB p65 mRNA was markedly downregulated (*p* < 0.05) in the LF and MF groups compared to the MC group (Figure 7B). Additionally, GAS17 mRNA expression was significantly (*p* < 0.05) reduced in the PC, LF, MF, HF, MS, and Q groups relative to the MC group (Figure 7C). Compared with the MC group, the mRNA expression levels of IL-1β, IL-8, and IL-2 were decreased in the PC, LF, MF, HF, MS, and Q groups (Figure 7D,F), while TNF-α mRNA expression was notably suppressed (*p* < 0.05) in the PC, MF, HF, MS, and Q groups (Figure 7G). Collectively, these findings indicate that sea buckthorn flavonoids may exert anti-inflammatory effects by inhibiting the expression of NF-κB p65, IL-1β, IL-2, IL-8, and TNF-α genes in the gastric tissues of *H. pylori*-infected mice. Moreover, sea buckthorn flavonoids may alleviate gastritis by suppressing GAS17 mRNA expression, thereby reducing excessive gastric acid secretion.

#### 3.4.5. Sea Buckthorn Flavonoids Changed Microbiota Composition in the Gastric Content

**Figure 8:**
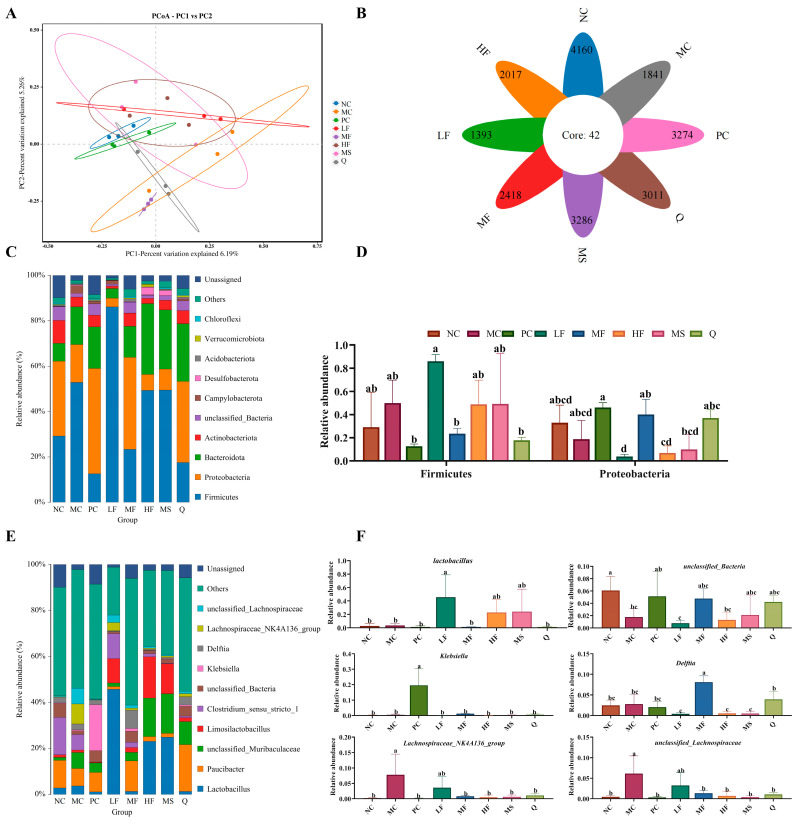

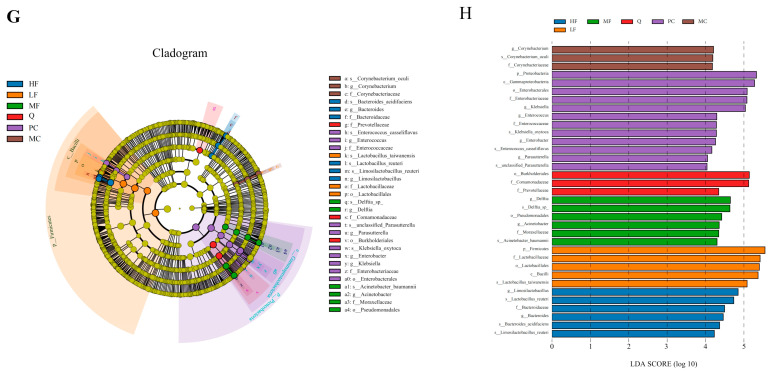
Effects of sea buckthorn flavonoids treatment on the structure and composition of the gut microbiota in *H. pylori* infected mice. (**A**) PCoA, (**B**) the flower diagram, (**C**) histogram of bacterial distribution at the phylum level, (**D**) relative abundance of Firmicutes and Proteobacteria, (**E**) histogram of bacterial distribution at the genus level, (**F**) significance analysis of bacterial abundance at the genus level, (**G**) cladogram showing the phylogenetic relationships of bacterial taxa. (**H**) Distribution histogram based on LDA, only the taxa with an LDA score higher than 4 are shown. Bars labeled with different letters (a, b, c, d) denote statistically significant differences at *p* ≤ 0.05, as determined by the Tukey-Kramer method.

We analyzed the changes in the gastric microbiota after intervention with sea buckthorn flavonoids using 16S rDNA sequencing (V3-V4 region). PCoA and flower figures showed that there were significant differences in the microbial community composition between the MC group and the sea buckthorn flavonoid intervention group (Figure 8A,B). At the phylum level, the gastric microbiota of mice was dominated by Firmicutes and Proteobacteria (Figure 8C,D). At the genus level, *Lactobacillus*, *Paucibacter*, *unclassified_Muribaculaceae*, *Limosilactobacillus*, *Clostridium_sensu_stricto_1*, *unclassified_Bacteria*, *Klebsiella*, *Delftia*, *Lachnospiraceae_NK4A136_group* and *unclassified_Lachnospiraceae* were the top 8 most abundant genera in the mouse gastric microbiota (Figure 8E). As can be seen from Figure 8F, the MC group displayed significant depletion of *unclassified_Bacteria* relative to the NC group, contrasting with concurrent elevations in *Lachnospiraceae_NK4A136_group* and *unclassified_Lachnospiraceae* taxonomic abundances (*p* < 0.05). Compared with the MC group, the treatment with sea buckthorn flavonoids and triple antibiotics led to a significant decrease in the abundance of *Lachnospiraceae_NK4A136_group* and *unclassified_Lachnospiraceae*. The abundance of *Lactobacillus* significantly increased in the LF group, the abundance of *Delftia* significantly increased in the MF group, and the abundance of *Klebsiella* significantly increased in the PC group (*p* < 0.05). In the MC, PC, Q, HF, LF, and MF groups, there were 5, 12, 3, 5, 4, and 5 species with significant differences (Figure 8G, *p* < 0.05), respectively. According to the evolutionary tree of the differential microbiota (Figure 8H), notable variations can be observed in the phylogenetic classification of the gut microbiota among the different mouse groups. Abundant bacterial taxa observed in the group MC were included *Corynebacterium*, *Corynebacterium oculi*, and *Corynebacteriaceae*. The bacterial taxa observed in the sea buckthorn flavonoids treatment groups included *Delftia*, *Pseudomonadales*, *Acinetobacter*, *Moraxellaceae*, *Acinetobacter baumannii*, Firmicutes, *Lactobacillaceae*, *Lactobacillales*, *Bacilli*, *Lactobacillus taiwanensis*, *Bacteroides acidifaciens*, Bacteroides, *Bacteroidaceae*, and *Lactobacillus reuteri*. The above results showed that sea buckthorn flavonoids could improve the structure of gastric microbiota in mice infected by *H. pylori* at phylum and genus levels and restored the composition of gastric microbiota to that in the normal mice.

## 4. Discussion

This study found that the main components of sea buckthorn flavonoids were rutin, quercetin-3-O-glucoside, quercetin, isorhamnetin, kaempferol-3-O-glucoside, kaempferol and epicatechin. Among them, quercetin, isorhamnetin, kaempferol and rutin have strong inhibitory activity against *H. pylori*. The order of antibacterial activity was: kaempferol (MIC = 19.54 μg/mL) > quercetin (MIC = 39.07 μg/mL) = isorhamnetin (MIC = 39.07 μg/mL) > rutin (MIC = 625 μg/mL). The differences in antibacterial activity may be related to the solubility and chemical structure of each component. The *H. pylori* cells in the quercetin treatment group showed obvious rupture, while in the isorhamnetin, kaempferol, mixed standard product and sea buckthorn flavonoid extract treatment groups, most of the cells shrank on the surface and changed from rod-shaped to spherical. Combined with the results of extracellular membrane permeability and content leakage tests, it is speculated that the flavonoid components may target the phospholipid bilayer of the cell membrane through hydrophobic interaction, thereby disrupting its fluidity and destroying the cell membrane structure, causing leakage of nucleic acids and proteins, and exerting antibacterial effects. This is consistent with the research results of previous studies [30]. Urease serves as a critical virulence factor that enables *H. pylori* to colonize the gastric mucosa. By generating a neutral microenvironment, urease facilitates *H. pylori* colonization in the acidic conditions of the stomach [31,34]. Kaempferol exhibits a more potent inhibitory effect on urease activity. It is hypothesized that kaempferol may suppress urease activity either by disrupting the cell membrane, leading to the leakage of enzymes or cofactors involved in urease production, or by directly interacting with the active site of urease, thereby interfering with its structural conformation or functional activity [35]. In conclusion, the bioactive flavonoids present in sea buckthorn demonstrate antibacterial activity against *H. pylori* through multiple mechanisms, including compromising cell membrane integrity, increasing outer membrane permeability, inducing leakage of intracellular contents, and inhibiting urease activity. Among these flavonoids, quercetin and isorhamnetin exhibit a more pronounced effect on disrupting the cell membrane at the same MIC, whereas kaempferol shows a stronger inhibitory effect specifically on urease activity.

The treatment groups of LF, MF and HF could down-regulate the mRNA expression levels of IL-2, IL-8,and IL-1β in the gastric tissues of *H. pylori*-infected mice. Meanwhile, it could reduce the contents of TNF-α and IL-1β in the serum of mice. The aforementioned findings are consistent with those of Lee et al., who demonstrated that menaquinone inhibits NF-κB pathway activation, thereby reducing the expression of pro-inflammatory cytokines (such as IL-1β, IL-6, IL-8, and TNF-α) in gastric tissue [36]. This study also revealed that sea buckthorn-derived flavonoids could elevate the levels of EGF in the gastric tissue of *H. pylori*-infected mice while simultaneously reducing the mRNA expression levels of GAS17. The up-regulation of EGF in the gastric mucosa may enhance the proliferation and regeneration of gastric mucosal cells. Meanwhile, Lin et al. demonstrated that chronic *H. pylori* infection may induce persistent inflammatory responses by activating the NF-κB signaling pathway and promoting the secretion of IL-8 [37]. Kim et al. reported that *H. pylori* infection can upregulate the expression of pro-inflammatory cytokines such as TNF-α, IL-8, and IL-1β both in vivo and in vitro through activation of the NF-κB pathway [38]. These pro-inflammatory cytokines not only serve as downstream effectors of NF-κB but can also further activate NF-κB signaling. Their expression in epithelial and inflammatory cells can be initiated by NF-κB-induced cytokines or through autocrine loops, potentially leading to the formation of a “cytokine network” that amplifies gastric mucosal inflammation [39]. Taken together, these findings indicate that sea buckthorn flavonoids may contribute to the suppression of gastric acid secretion, the mitigation of gastric mucosal injury, and the promotion of mucosal repair.

Studies have shown that *H. pylori* infection can disrupt the microecology of the stomach, leading to dysbiosis of the gastric microbiota [40]. In our study, significant differences in the composition of the gastric microbial community were observed between the *H. pylori* infection group and the sea buckthorn flavonoid intervention group. Notably, the abundance of *Lactobacillus* was markedly higher in the LF group compared to the MC group. *Lactobacillus* demonstrates antibacterial activity against pathogens in both the gastrointestinal and urinary tracts and has been found to enhance the production of IL-10 through the TLR2 signaling pathway [41,42]. Therefore, the observed increase in *Lactobacillus* abundance suggests that low-dose sea buckthorn flavonoid treatment may contribute to the restoration of gastric microbiota balance.

Taken together, sea buckthorn flavonoids may exert therapeutic effects on *H. pylori*-induced gastritis through multiple mechanisms, such as inhibiting the NF-κB pathway to reduce inflammation, promoting the repair of gastric mucosa, and restoring the balance of gastric microbiota. This study lays a theoretical foundation for the adjuvant treatment of *H. pylori* infection with plant active substances. Based on the multiple activities of sea buckthorn flavonoids (antibacterial, anti-inflammatory, and gastric mucosa repair effects), functional health products targeting gastrointestinal health can be developed. Meanwhile, it provides references for plant-derived candidate molecules and action targets for the research and development of new anti-*H. pylori* drugs. However, this study still has significant limitations. The current research conclusions mainly rely on in vitro antibacterial experiments and data from mouse models, lacking direct evidence from human clinical trials. The pharmacokinetic characteristics, bioavailability, and safety of the subject in humans remain unclear.

## 5. Conclusions

The potential anti-*H. pylori* activity of flavonoids in sea buckthorn was exploratively investigated. The primary flavonoid components of sea buckthorn include rutin, quercetin, quercetin-3-O-glucoside, isorhamnetin, kaempferol 3-O-glucoside, and kaempferol. Among these, quercetin, isorhamnetin, and kaempferol exhibit potent inhibitory effects against *H. pylori*. When the concentrations exceed the MIC, the inhibition rate of *H. pylori* exceeds 50%. Furthermore, as the concentration increases, the antibacterial activity, cell membrane permeability, and nucleic acid and protein concentrations all show a gradual upward trend. Sea buckthorn flavonoids could inhibit the growth of *H. pylori* by inducing morphological changes from rod-shaped to spherical, leading to cellular shrinkage and rupture. Meanwhile, these flavonoids disrupt the bacterial cell membrane, resulting in the leakage of macromolecules, increased membrane permeability, and inhibition of urease activity. In vivo experiments have demonstrated that extracts of sea buckthorn flavonoids can ameliorate *H. pylori*-induced gastritis in mice through multiple mechanisms, including inhibition of the NF-κB signaling pathway, down-regulation of GAS17 to suppress gastric acid secretion, up-regulation of EGF to promote gastric mucosal regeneration, and modulation of the gastric microbiota.

## Figures and Tables

**Table 1:** The main flavonoid composition in sea buckthorn extract.

Flavonoids	Molecular Weight	Molecular Formula	Concentration (ng/mg)
3,4-Dihydroxybenzoic acid	153.02	C_7_H_6_O_4_	192.84 ± 7.95
Catechin	289.07	C_15_H_14_O_6_	72.21 ± 3.22
Kaempferol-3-O-glucoside	447.09	C_21_H_20_O_11_	41.04 ± 0.08
Kaempferol	285.04	C_15_H_10_O_6_	21.11 ± 0.14
Epicatechin	289.07	C_15_H_14_O_6_	24.33 ± 0.91
Rutin	609.15	C_27_H_30_O_16_	433.01 ± 7.33
Quercetin 3-β-D-glucoside	463.09	C_21_H_20_O_12_	215.39 ± 5.91
Isorhamnetin	315.05	C_16_H_12_O_7_	114.42 ± 2.16
Quercetin	301.04	C_15_H_10_O_7_	191.9 ± 1.05

**Table 2:** 16S rDNA sequence alignment results.

Sequence Number	Bacterial Scientific Name	Similarity	Description
1	*Helicobacter pylori*	100%	ATCC 43504
2	*Helicobacter pylori*	99.93%	NCTC 11637
3	*Helicobacter cetorum*	97.89%	MIT 99-5656

## Data Availability

The original contributions presented in this study are included in the article/Supplementary Materials. Further inquiries can be directed to the corresponding author.

## Supplementary Materials

The following supporting information can be downloaded at: https://www.mdpi.com/article/10.3390/foods14233995/s1, Table S1: Primer sequence of gene.
