# Supplementary material for: Inhibitory Effects and Mechanisms of Flavonoids in Sea Buckthornon (Hippophae rhamnoides L.) on Helicobacter pylori
Source: Foods. 2025 Nov 21;14(23):3995. doi: 10.3390/foods14233995 (PMC12691763; doi:10.3390/foods14233995)
Supplement: Supplementary file 1 [file foods-14-03995-s001.zip › foods-3987973-supplementary.pdf]

Table S1 Primer sequence of gene

Gene	Primer sequences (5' to 3')
β-actin	F: GTATGAAGGCTTTGGTCTC<br>R: TTGGTCTCAAGTCAGTGTA
GAS17	F: GCATCACCGTCTCTCAGGTT<br>R: TGTTCGCCTCCTCTTGGAAC
IL-2	F: TCAGCAACTGTGGTGGACTTT<br>R: TGAGGGCTTGTTGAGATGATGC
TNF-α	F: ATGGCCTCCCTCTCATCAGT<br>R: GGCTACAGGCTTGTCACTCG
NF-κB p65	F: AGTATTCCTGGCGAGAGA<br>R: AACTGTTCCTGGTCCTGT
IKB-α	F: CAGTGTAGCAGTCTTGACG<br>R: GATAGAGGCTAGGTGCAGA
IL-8	F: ATGGCTGCTCAAGGCTGGTC<br>R: TCACAGGGACGGACGAAGAT
IL-1β	F: GGAAGTCTGTCTGCTCAGTATT<br>R: CGTCAACTTCAAAGAACAGGT
